# *Withania somnifera* and *Centella asiatica* Extracts Ameliorate Behavioral Deficits in an In Vivo *Drosophila melanogaster* Model of Oxidative Stress

**DOI:** 10.3390/antiox11010121

**Published:** 2022-01-06

**Authors:** Kadine Cabey, Dani M. Long, Alexander Law, Nora E. Gray, Christine McClure, Maya Caruso, Parnian Lak, Kirsten M. Wright, Jan F. Stevens, Claudia S. Maier, Amala Soumyanath, Doris Kretzschmar

**Affiliations:** 1 BENFRA Botanical Dietary Supplements Research Center, Oregon Health and Science University, Portland, OR 97239, USA; cabey@ohsu.edu (K.C.); longdan@ohsu.edu (D.M.L.); law@ohsu.edu (A.L.); grayn@ohsu.edu (N.E.G.); mcclurch@ohsu.edu (C.M.); wrigkir@ohsu.edu (K.M.W.); soumyana@ohsu.edu (A.S.); 2 Department of Neurology, Oregon Health and Science University, Portland, OR 97239, USA; carusma@ohsu.edu; 3 Helfgott Research Institute, National University of Natural Medicine, Portland, OR 97201, USA; 4 Oregon Institute of Occupational Health Sciences, Oregon Health and Science University, Portland, OR 97239, USA; 5 Department of Chemistry, Oregon State University, Corvallis, OR 97331, USA; plak@berkeley.edu (P.L.); claudia.maier@oregonstate.edu (C.S.M.); 6 Linus Pauling Institute, Oregon State University, Corvallis, OR 97331, USA; fred.stevens@oregonstate.edu; 7 College of Pharmacy, Oregon State University, Corvallis, OR 97331, USA

**Keywords:** ashwagandha, gotu kola, cognition, sleep, oxidative stress

## Abstract

Due to an increase in the aging population, age-related diseases and age-related changes, such as diminished cognition and sleep disturbances, are an increasing health threat. It has been suggested that an increase in oxidative stress underlies many of these changes. Current treatments for these diseases and changes either have low efficacy or have deleterious side effects preventing long-time use. Therefore, alternative treatments that promote healthy aging and provide resilience against these health threats are needed. The herbs *Withania somnifera* and *Centella asiatica* may be two such alternatives because both have been connected with reducing oxidative stress and could therefore ameliorate age-related impairments. To test the effects of these herbs on behavioral phenotypes induced by oxidative stress, we used the *Drosophila melanogaster sniffer* mutant which has high levels of oxidative stress due to reduced carbonyl reductase activity. Effects on cognition and mobility were assessed using phototaxis assays and both, *W. somnifera* and *C. asiatica* water extracts improved phototaxis in *sniffer* mutants. In addition, *W. somnifera* improved nighttime sleep in male and female *sniffer* flies and promoted a less fragmented sleep pattern in male *sniffer* flies. This suggests that *W. somnifera* and *C. asiatica* can ameliorate oxidative stress-related changes in behavior and that by doing so they might promote healthy aging in humans.

## 1. Introduction

Throughout the world, many countries are experiencing population aging, a phenomenon where the portion of older persons in a population increases while that of younger persons decreases [1]. Reports from the United Nations [1] and the United States Census Bureau [2] predict that the number of persons aged 60–65 years or older globally will increase to 1.6–2 billion persons in 2050 and 2.4 billion persons in 2100. Population ageing is thought to be due mainly to a decline in fertility rate and increase in longevity [1,2]. Among the concerns associated with an increase in population aging is the prevalence of age-related disease, i.e., diseases or conditions that occur more frequently with advanced age, and the economic and social impact of associated health care. Age-related diseases include cancer, type 2 diabetes mellitus, cardiovascular disease, musculoskeletal disorders, arthritis and neurodegenerative diseases like Parkinson’s disease and Alzheimer’s disease [3,4,5]. In addition to age-related diseases, aging is also associated with non-pathological changes in cognition [6,7], sleep [8,9,10] and mood [11,12].

Cognitive decline can be treated pharmacologically using cholinesterase inhibitors [13,14] and non-pharmacologically using computer-assisted cognitive training, exercise and a variety of psychosocial group interventions [15]. Evidence for the efficacy of cholinesterase inhibitors to reduce the rate of cognitive decline is mixed. One systematic review reported that cholinesterase inhibitors did not improve cognition or reduce the incidence of dementia in persons with mild cognitive impairment [15]. A more recent systematic review and meta-analysis reported that cholinesterase inhibitors did not improve cognition but were associated with a decrease in progression to dementia [16]. Cholinesterase inhibitors were also found to have notable side effects including dizziness, headache, insomnia and diarrhea [16]. Non-pharmacological treatments of mild cognitive impairment were generally found to have no benefits except for family and heterogenous psychosocial interventions. However, only one, low-quality study of each psychosocial intervention was evaluated [15].

Sleep changes in aging can also be treated pharmacologically and non-pharmacologically. Pharmacological treatments include melatonin, trazodone, benzodiazepines and non-benzodiazepine hypnotics [8]. Pharmacological treatments are not preferred as first-line treatment in older populations due to side effects including dizziness and hypotension and risk of falls. Some drugs, such as benzodiazepines, are only recommend for short term use of up to 4 weeks [8,9]. Sleep disturbances can be treated non-pharmacologically using cognitive behavioral therapy, sleep restriction, sleep stimulus control and sleep hygiene education [8,9,10]. Non-pharmacological approaches are preferred as first-line strategies and some treatments such as cognitive behavioral therapy have been seen to be effective in treating insomnia in older populations [9] while others such as sleep hygiene have not been shown to be effective for treating insomnia when used alone [10].

Given the increase in the size of the aging population and consequently age-related diseases or functional changes, there is a need to identify safe and effective treatments for symptoms, in particular treatments which address multiple related symptoms. Two such potential treatments are the herbs *Withania somnifera* (L.) Dunal (family Solanaceae) and *Centella asiatica* (L.) Urban (family Apiaceae).

*W. somnifera*, more commonly known as ashwagandha, winter cherry, Indian ginseng and asgand [17,18,19], is a small woody shrub used for its medicinal properties in several traditional healing systems including Ayurveda, Unani and Siddha [17,18,20]. The dried roots of the plant are mainly employed in treatment, but the leaves, flowers and seeds are also utilized to a lesser extent [17,19]. *W. somnifera* is traditionally used to treat numerous conditions including asthma, arthritis, anxiety, sleep disorders and ulcers [17,19]. In Ayurveda this plant is regarded as a rasayana herb, i.e., one which promotes health and longevity, slows down ageing and increases the body’s resistance to disease and fatigue [17,20]. The unique specialized metabolites of *W. somnifera*, often regarded as the active compounds, are known collectively as withanolides, of which over 50 derivatives have been identified [21].

*C. asiatica* is a slender, usually creeping plant that has also been used in traditional healing [22,23]. *C. asiatica* has several aliases such as gotu kola, brahmi, Indian pennywort, buak bok and kaki kuda [22,23]. *C. asiatica* has been used traditionally in Chinese medicine and Ayurvedic medicine to treat skin conditions including leprosy, and eczema as well as dysentery and gastric ulcers [22,24,25]. Like *W. somnifera*, *C. asiatica* is regarded as a rasayana herb that rejuvenates, boosts memory, prevents cognitive deficits and improves brain function [25]. Two groups of compounds associated with the activity of *C. asiatica* include the herb’s triterpene (TT) and caffeoylquinic acid (CQA) components [25].

Age-related diseases and impairments have often been connected with oxidative stress. Oxidative stress, defined as an imbalance between reactive oxygen species (ROS) and antioxidant systems resulting in excess ROS, is associated with degenerative changes in cells and tissues both in normal aging and age-related diseases [26,27]. High levels of ROS can cause damage to DNA, proteins and lipids impairing their function [4,27]. Numerous studies have investigated the therapeutic effects of *W. somnifera* and *C. asiatica*. In vivo studies have shown that both of these herbs exhibit antioxidant properties with *W. somnifera* increasing the expression levels of antioxidant enzymes [28] and water extracts of *C. asiatica* activating the the transcription factor nuclear erythroid factor 2 (Nrf2), a master regulator of the antioxidant response element [29]. Studies have also shown that *W. somnifera* and *C. asiatica* exhibit cognitive protecting properties [28,29] and that *W. somnifera* exhibits sleep-promoting properties [30]. However, the ability of these botanicals to provide resilience to age-related changes in cognition and sleep in connection with their antioxidant properties had not been fully evaluated.

*Drosophila melanogaster,* commonly known as the fruit fly, has been used as a model organism in biomedical research for more than a century to investigate biological processes including genetics and inheritance, aging, learning and behavior [31,32,33]. *D. melanogaster* serves as an efficient in vivo model as it has a short developmental cycle of about 10 days and an adult lifespan of about 60 days, produces numerous offspring, possess genes with sequences and functions preserved in mammals, is relatively easy and economical to maintain, and can provide a model in which the environment and genetic background can be controlled [31,32,33]. In selecting a *D. melanogaster* mutant to investigate oxidative stress, the mutant *sniffer* can be used. The *sniffer* gene in *D. melanogaster* codes for a short-chain dehydrogenase/reductase enzyme that shows carbonyl reductase activity [34]. In humans, carbonyl reductases help mitigate the effects of oxidation of molecules like lipids [35]. Given that the primary structure of the *sniffer* coded carbonyl reductase in *D. melanogaster* is very similar to that of humans [35], mutations in this gene in the fruit fly can be used to replicate high levels of oxidative stress in humans and the impacts of those high levels of oxidative stress. Indeed, *sniffer* flies exhibit reduced lifespan, age-related neurodegeneration and a sluggish walking phenotype that worsens with age [34].

Given the need for alternative treatments for addressing oxidative stress, cognitive decline and disturbed sleep in normal and pathological aging, and the pre-clinical evidence of these herbs in treating these symptoms, the cognitive and sleep promoting ability of *W. somnifera* and *C. asiatica* was evaluated in this *D. melanogaster* model of oxidative stress.

## 2. Materials and Methods

### 2.1. Fly Stocks

*D. melanogaster* wild-type Canton S (CS) and mutant *sniffer* flies were used in these investigations. CS flies were originally provided by M. Heisenberg, University of Würzburg, Germany, *sniffer* flies were isolated by D. Kretzschmar and J. Botella-Munoz at the University of Regensburg, Germany as described by Botella at al. [34]. Briefly, viable flies were collected following P-element insertions and flies with an insertion into the *sniffer* gene were identified. Flies were maintained on standard *Drosophila* food in a 12 h light and dark chamber at 25 °C.

### 2.2. Interventions

#### 2.2.1. Raw Plant Materials

Dried root of *W. somnifera* plants grown at Oregon’s Wild Harvest (OWH), Redmond, OR, USA (OWH lot number 201000162) was obtained in bulk. Voucher samples of the root have been deposited in the Oregon State University (OSU) Herbarium (voucher number OSC-V-265405) as well as retained in our laboratories (Code number BEN-WS-8) at Oregon Health and Science University (OHSU).

Dried plant material of *C. asiatica* (OWH Lot# GOT-03193c-OHQ, OSU Herbarium Voucher# OSC-V-258627) was purchased in bulk through Oregon’s Wild Harvest, Redmond, OR, USA). A sample is retained in our OHSU laboratories under the OWH Lot number.

#### 2.2.2. Preparation of Extracts

Aqueous extracts of *W. somnifera* dried ground roots and *C. asiatica* dried aerial parts were prepared by boiling the plant material in deionized water under reflux for 90 min at a ratio of 160 g material: 2 L water. The mixtures were filtered through a kitchen sieve while still warm, to remove larger plant pieces. Sieved extracts were centrifuged at 3750 rpm for 10 min on a benchtop centrifuge (Beckman GS-6R) to sediment finer particles and the supernatant filtered through Whatman filter paper (Grade 1, 90 mm). The filtered extract was then frozen and lyophilized into a powder on a Virtis lyophilizer (Phase 1, 115 V, 20 amps). Extractions were repeated as needed, the dried extracts were given a specific lot number and stored at −20 °C until use. *W. somnifera* extract (BEN-WS-Aq-9) is stored in our laboratory at OHSU. This extract will be referred to as “WS” in the remainder of the text. Several batches of the *C. asiatica* extract made from the sample plant material were blended to give “CAW MIX” of which a voucher sample is stored in our laboratory at OHSU. This will be referred to as “CAW” in the remainder of the text. A detailed, untargeted analysis of this complex extract by liquid chromatgraphy coupled to high resolution mass spectrometry has been recorded and archived at the Mass Spectrometry Center, Oregon State University, and will be made available on request to the corresponding author.

#### 2.2.3. Fractionation of CAW

Powdered CAW (140.5 g) was shaken with methanol (3 × 200 mL). The solid, methanol insoluble portion of the resulting extract was collected by filtration and dried under the fume hood at room temperature (A4; 41.3 g). The combined methanol soluble portions (600 mL) were dried under vacuum. The dry-sticky residue obtained after evaporation was partitioned between water (200 mL) and dichloromethane (DCM; 200 mL). The DCM layer was washed with additional water aliquots (2 × 200 mL) and then dried under vacuum (A3; 7.83 g). The total water phase (600 mL) was then extracted with butanol (BuOH; 3 × 600 mL). The water layer (600 mL) was separated and dried by lyophilization (A2; 68.2 g). The butanol layer (1800 mL) was collected and dried under vacuum (A1; 15.95 g; 11.35% of original CAW).

Of the four fractions A1–A4, A1 was found to have the strongest protective activity against intracellular Aβ toxicity in MC65 neuroblastoma cells (data not shown), a model in which CAW also shows protection. A1 was therefore tested for its effects on phototaxis in *sniffer* flies.

#### 2.2.4. Analysis of A1 and Preparation of Matching CQA and TT Mixtures

High performance liquid chromatography with UV Detection (HPLC-UV) was used to determine the concentration of CQAs and TTs in CAW and A1.

Chromatography was performed on an Agilent 1100 quaternary gradient HPLC with photodiode detector using an Eclipse Plus C8 column (4.6 × 150 mm, 3.5 µm) with Eclipse Plus C8 guard (4.6 × 12.5 mm, 5 µm) and a stepwise gradient of acetontrile and water both containing acetic acid (0.05%). Acetonitrile concentration increased from 5% to 20% from minute 0 to 6, then from 20% to 40% between minute 12 and 13, rising to 90% by minute 14. After holding at 90% until minute 17, the % returned to 5% at minute 17.2 for re-equilibration until minute 20. Detection of CQAs and TTs was done at 330 nm and 205 nm respectively. The concentration of compounds in CAW and A1 (Table 1) was obtained by comparison of peak areas to co-chromatographed solutions of standard CQA and TT compounds (ChromaDex, Irvine, CA, USA).

#### 2.2.5. Preparation of Test Food for *D. melanogaster* Flies

For stock solutions, CAW was dissolved in water (100 mg/mL) and subfraction A1 was dissolved in ethanol (11.35 mg/mL). A mix of caffeoylquinic acids (CQAs) equivalent to those found in A1 was created by dissolving commercially available pure compounds (Chromadex, Irvine, CA, USA) in ethanol (Table 1). A mix of triterpenes known as *Centella asiatica* selected triterpenes (CAST^TM^; a gift from Indena SpA, Milan, Italy) was dissolved in ethanol (2 mg/mL) to provide similar (but not identical) concentration of TTs to those found in A1 (Table 1). WS was dissolved in water (10 or 40 mg/mL). Stock solutions of CAW, A1, CQAs, CAST and WS were diluted into standard *Drosophila* food to give the final desired test concentrations. For the yeast paste, stock solutions were diluted in 10% baking yeast solution (Fleischmann’s Dry Yeast in water). Control diets were prepared by diluting the appropriate solvent (water or ethanol) into standard *Drosophila* food or yeast paste.

#### 2.2.6. Administration to *D. melanogaster* Flies

Newly eclosed flies were collected daily from stock vials to obtain flies not older than 24 h. They were then transferred to fresh vials containing standard *Drosophila* food (controls) or standard *Drosophila* food supplemented with extracts of *W. somnifera* (WS; 0.5, 2 mg/g) or *C. asiatica* (CAW; 1 or 10 mg/g). After 7 days, flies were separated by experimental group and sex and tested in groups of 10–20 flies in the fast phototaxis assay (both treatments) or used in sleep experiments (WS only). Sex was determined by the sexual dimorphic pattern on the abdomen.

We also tested the influence of CAW subfraction A1 and the CQA and CAST^TM^ compound mixes on phototaxis in *sniffer* flies. However, as the quantities of these test materials was limited, we changed the administration method to mixing CAW, subfraction A1, or CQA and CAST^TM^ compound mixes in yeast paste (see Section 2.2.4) and providing 500 µL of this as a layer on top of standard *Drosophila* food (to prevent drying out of the yeast paste) rather than incorporating the materials in the food itself.

### 2.3. Effects of WS Extract on NRF2 Activation in HepG2-ARE Reporter Cells

HepG2 cells that stably express a firefly luciferase gene under the control of the antioxidant response element (ARE) promoter were obtained from BPS Bioscience. Cells were grown in MEM medium supplemented with FBS (10%), non-essential amino acids (1%), 1 mM sodium pyruvate (1 mM) and penicillin/streptomycin (1%). For the assay, cells were plated at a density of 30,000 per well in a 96-well plate and treated for 48 h with WS (50 ug/mL) or CAW (100 µg/mL). NRF2 activation was then quantified using the Pierce Firefly Luc One-Step Glow Assay Kit (ThermoFisher Scientific, Waltham, MA, USA) as per the manufacturer’s instructions. Luminescence was normalized to total protein content as determined by a bicinchoninic acid (BCA) assay (Pierce^TM^ BCA Protein Assay Kit, ThermoFisher Scientific, Waltham, MA, USA).

### 2.4. Phototaxis

Experiments were performed to compare the phototaxis response of untreated *sniffer* flies to that of untreated wild type CS flies, as well as *sniffer* flies treated with, CAW, A1, CQAs, CAST (TTs) or WS. Fast phototaxis assays were conducted in the dark as previously described in Dutta et al. [36] and Bolkan et al. [37] using the countercurrent apparatus described by Benzer [38] and a single light source. A detailed description of the experimental conditions can be found in studies by Strauss and Heisenberg [39]. After 7 days on control or treatment diets, flies were allowed to transition towards the light in 5 consecutive runs lasting 6 s each. Flies were then scored based on the tube they were contained in at the end of the final run.

### 2.5. Sleep Experiments

Sleep patterns in male and female CS and *sniffer* flies aged 1 week were assessed using the *Drosophila* Activity Monitoring Systems (DAMS) as described by Cassar et al. [40] and Metaxakis et al. [41]. For 7 days prior to placement in the monitors, CS flies were fed standard *Drosophila* food while *sniffer* flies were fed either standard *Drosophila* food or standard food supplemented with WS (0.5 mg/g or 2 mg/g). When placed in the activity monitor, all flies were aged 8 days. Flies were placed individually in glass tubes with standard *Drosophila* food placed on one end and the other end sealed with a short piece of yarn (approx. 1.5 cm). Glass tubes were placed in DAMS model DAM2 (Trikinetics, Waltham, MA, USA).

Flies were left in the activity monitor for 9 days. Locomotor activity of the flies was analysed once every minute for 7 days of light/dark cycles (12 h:12 h LD). Data from the first day were not included to allow the flies to get used to the new environment and the flies were removed from the monitor on day 9. A sleep bout was regarded as a period of 5-min or more with no movement detected.

### 2.6. Statistical Analyses

Phototaxis data were analysed using GraphPad (v.5 for Windows, San Diego, CA, USA) for each sex in each intervention type. A Kruskal–Wallis test with a post-Dunn’s multiple comparison test was used to compare locomotor activity between more than 2 groups and a Mann–Whitney test was used to compare locomotor activity between 2 groups. Sleep data were analysed using ClockLab (v.6.1.02 for Windows, Actimetrics, Wilmette, IL, USA). A 2-tailed unpaired *t*-test with Welch’s correction was done to compare sleep activity within experimental groups and a one-way ANOVA was done to compare experimental groups to a control (untreated flies). A *p*-value of <0.05 was considered significant for all analyses.

## 3. Results

### 3.1. NRF2 Activation

**Figure 1:**
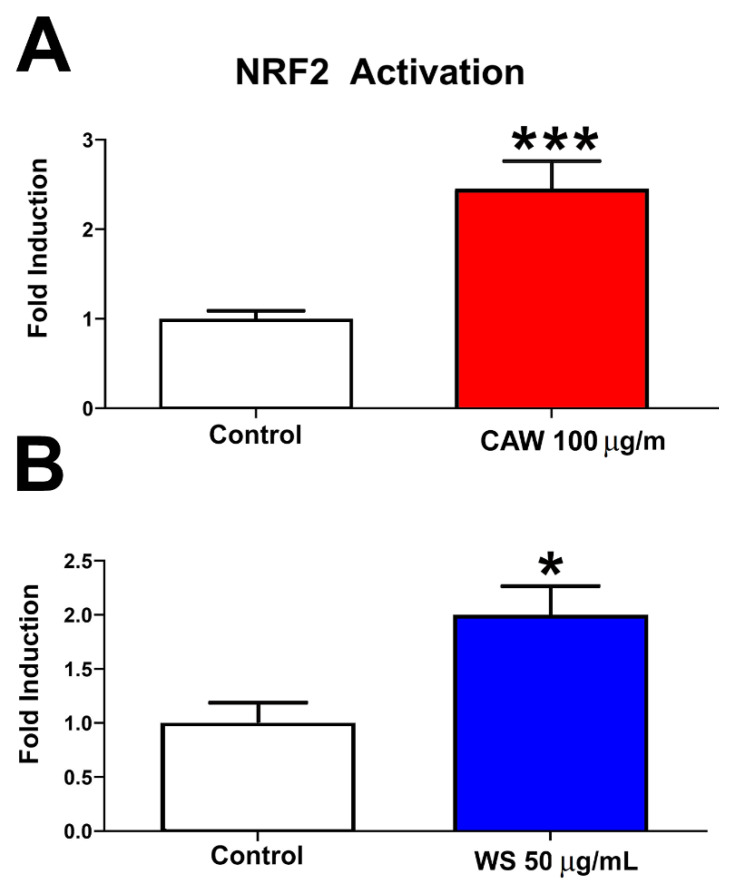
NRF2 activity in the presence and absence of *W. somnifera* water extract (WS) or *C. asiatica* water extract (CAW). HepG2-ARE cells treated with CAW (**A**) WS (**B**) showed a significant increase in NRF2 activity compared to controls (* = *p* < 0.05, *** = *p* < 0.001). Eighteen independent measurements over 3 replicate experiments were performed. Error bars represent standard error of the mean.

As mentioned in the introduction, we aimed to test whether anti-oxidative effects mediated by *W. somnifera* and *C. asiatica* ameliorate behavioral deficits in a *Drosophila* model. We previously showed that treatment with our *C. asiatica* water extract increased the expression of mitochondrial and antioxidant response genes in mice and hippocampal neurons exposed to beta amyloid (Aβ). This included an increase in nuclear factor erythroid-derived 2 (NRF2), a key factor in regulating the response to oxidative stress by inducing the expression of several genes involved in mitigating oxidative stress [42,43,44]. Effects of *C. asiatica* on oxidative stress have also been described by several other groups using different methods [24,45]. A role of *W. somnifera* in reducing oxidative stress is less well documented although several papers suggest such a function [46,47,48]. To confirm that our *C. asiatica* and *W. somnifera* extracts promote oxidative stress responses, we measured NRF2 activity in a reporter assay using HepG2-ARE cells. Treating cells with 50 µg/mL WS or with 100 µg/mL CAW did significantly increase NRF2 activity in this assay (Figure 1).

### 3.2. Phototaxis

#### 3.2.1. *Sniffer* Flies Show Reduced Performance in the Phototaxis Test Compared to CS Wild Type Flies

**Figure 2:**
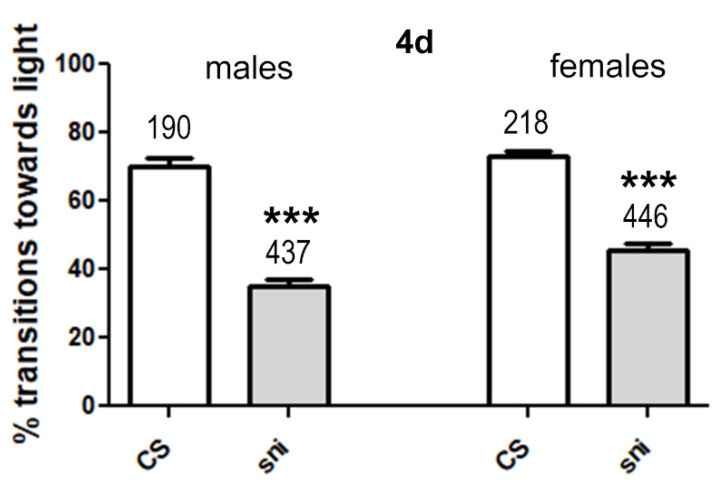
Percentage transitions towards light for *sniffer* (sni) and Canton S (CS) flies. *Drosophila melanogaster sniffer* mutants and CS flies were fed standard food for 4 days. Fast phototaxis was then performed with groups of 10–20 flies separated by genotype and sex. The number of flies tested is indicated above the bars. Error bars represent SEMs *** = *p* < 0.001.

To validate the model, we tested whether *sniffer flies* (sni) showed a difference in their performance in the fast phototaxis test when compared to CS flies. Both, male and female *sniffer* flies fed standard *Drosophila* food showed significantly lower transitions towards the light than CS flies even when only 4 days old (Figure 2). This shows that *sniffer* flies are significantly impaired in their performance in this assay, which tests locomotion and the ability of the flies to detect and orient towards light. This confirmed that we can use this assay to determine beneficial effects of CAW and WS on the performance of this oxidative stress-related mutant fly model.

#### 3.2.2. CAW at Higher Concentrations in *Drosophila* Food Improves Phototaxis in Female *Sniffer* Flies

**Figure 3:**
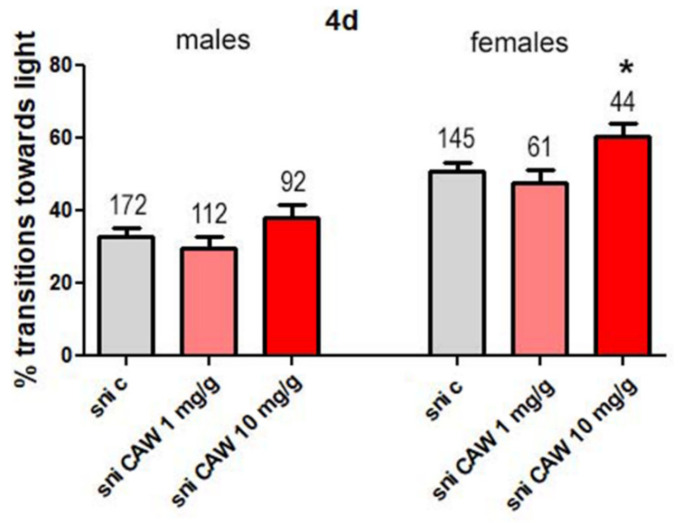
Percentage transitions towards light for *sniffer* (sni) flies treated with *Centella asiatica* water extract (CAW; 1 mg/g and 10 mg/g). *Sniffer* mutants were fed standard food (c) or standard food supplemented with CAW (1 mg/g or 10 mg/g) for 4 days. Fast phototaxis was performed with flies in groups of 10–20 flies and separated by experimental group and sex. The number of flies tested is indicated above the bars. Error bars represent SEMs. * = *p* < 0.05.

We then tested different concentrations of CAW (1 or 10 mg/g) mixed with *Drosophila* food to determine effects on phototaxis. There were no significant differences seen in transitions towards light between control male *sniffer* flies and flies treated with CAW 1 mg/g or 10 mg/g (Figure 3).

With female *sniffer* flies the difference in transitions towards the light between control flies and treated flies was not significant at 1 mg/g CAW but there was significant improvement with 10 mg/g CAW (Figure 3).

#### 3.2.3. CAW Mixed into Yeast Paste Improves Phototaxis in *Sniffer* Flies

As described in the next section, we also intended to test a subfraction and compounds from CAW. However, the amount available of these substances was limited and we therefore wanted to establish a feeding paradigm that required less material. We therefore tested the effect of applying CAW in yeast paste, which was layered on top of the regular food, rather than incorporating CAW into the food.

**Figure 4:**
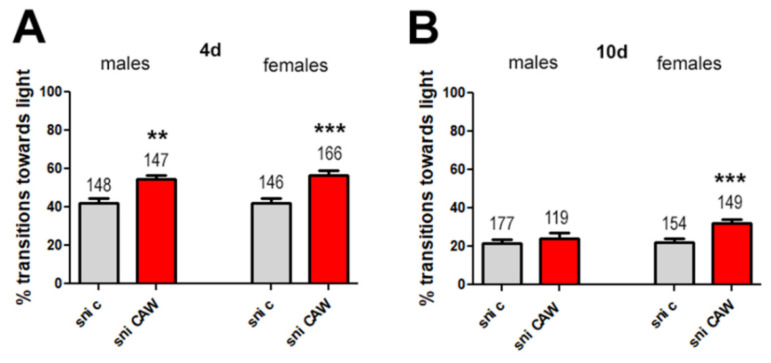
Percentage transitions towards light for *Sniffer* (sni) flies treated with CAW (10 mg/g) provided in yeast paste. *Drosophila melanogaster sniffer* mutants were fed yeast paste (c) or yeast paste supplemented with CAW (10 mg/g) for 4 days (**A**) or 10 days (**B**). Fast phototaxis was performed with flies in groups of 10–15 flies and separated by experimental group and sex. The number of flies tested is indicated above the bars. Error bars represent SEMs. ** = *p* < 0.01, *** = *p* < 0.001.

When CAW was administered in yeast paste at 10 mg/g, a significant difference in transition towards the light was now seen between untreated and treated male and female *sniffer* flies when4 day old (Figure 4A).

In addition to the *sniffer* flies aged to 4 days, we also tested 10 day old flies to determine whether treatment is still beneficial at a stage where the behavioral phenotype is more advanced. As seen in Figure 4B, the performance of *sniffer* flies worsens with age with flies aged 10 days only transitioning at 22% whereas the rate is 42% in 4 days old flies (Figure 4A). At this advanced stage, no significant difference in transition towards the light was observed between male flies treated with 10 mg/g CAW and male control flies (Figure 4B). However, a significant difference in transition towards the light was still noted between female *sniffer* flies and female control flies aged 10 days (Figure 3).

#### 3.2.4. CAW A1 Fraction and CQAs Improve Phototaxis in *Sniffer* Flies

**Figure 5:**
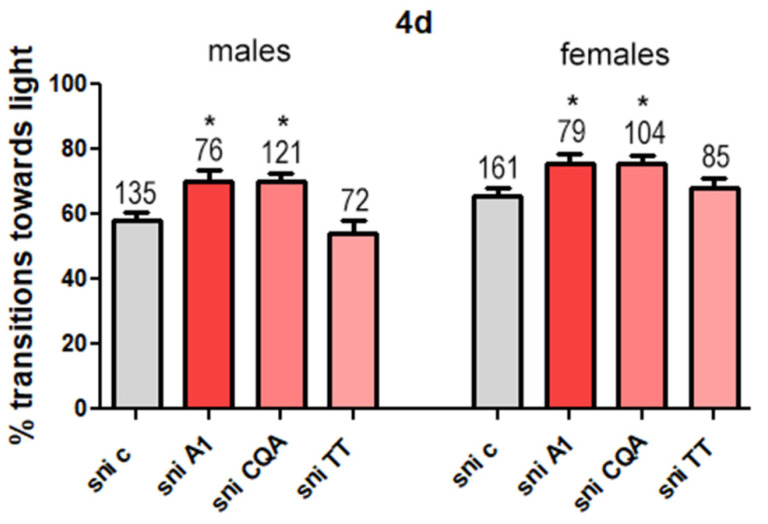
Percentage transitions towards light for *sniffer* (sni) flies treated with CAW subfraction A1, caffeoylquinic acids (CQAs) and CAST triterpenes (TTs). *Sniffer* mutants were fed yeast paste (c) or yeast paste supplemented with subfraction A1, CQAs or TTs for 4 days. Fast phototaxis was performed with flies in groups of 10–20 flies and separated by experimental group and sex. The number of flies tested is indicated above the bars. Error bars represent SEM. * = *p* < 0.05.

In an attempt to identify active compounds in CAW, we first tested a subfraction (A1) that had shown protective effects against Aβ-induced toxicity in neuroblastoma cells (data unpublished) and was enriched in CQAs and TTs compared to CAW. We found that both male and female *sniffer* flies showed significantly greater transitions towards the light when treated with A1 (Figure 5) than control flies. Due to this positive result, we then tested a CQA mix and CAST, a TT mix, which contained these compounds at similar concentrations to those found in A1 (Table 1).

Whereas the treatment with the CQA mix ameliorated the behavioral deficits in both sexes compared to control flies, the difference in transition towards the light between control flies and those treated with the TT mix was not significant in either sex (Figure 5).

#### 3.2.5. WS Improves Phototaxis in *Sniffer* Flies

**Figure 6:**
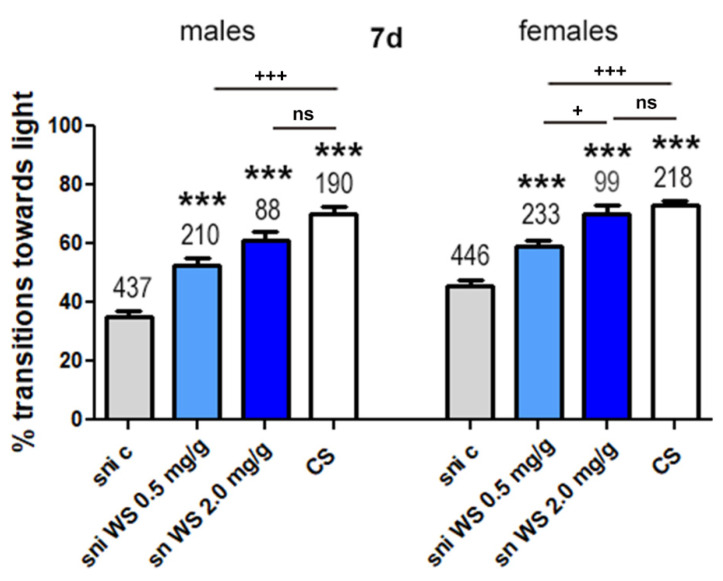
Percentage transitions towards light for *sniffer* (sni) flies treated with *Withania somnifera* water extract (WS; 0.5 mg/g and 2 mg/g). *Drosophila melanogaster sniffer* mutants were fed standard food (c) or standard food supplemented with WS (0.5 mg/g or 2 mg/g) for 7 days. Fast phototaxis was performed with flies in groups of 10–20 flies and separated by experimental group and sex. The number of flies tested is indicated above the bars. Error bars represent SEM. *** = *p* < 0.001 compared to sni control, + = *p* < 0.05, +++ = *p* < 0.001 comparing the two indicated bars. ns = not significant.

In a second set of experiments, we tested effects of *W. somnifera* water extract (WS) on behavioral phenotypes induced by the *sniffer* mutation. Male and female *sniffer* flies treated with WS (0.5 mg/g and 2 mg/g) saw a significant increase in transitions towards light compared to control *sniffer* flies (Figure 6). While treatment with WS 0.5 mg/g ameliorated the effects on performance, the flies were still significantly worse than CS. In contrast, when using 2 mg/g the flies showed such a strong performance that they were not significantly different from CS flies (Figure 6).

### 3.3. Sleep

We then tested whether WS has a beneficial impact on sleep in *sniffer* flies due to the well-known sleep-promoting properties of *W. somnifera* [30].

**Figure 7:**
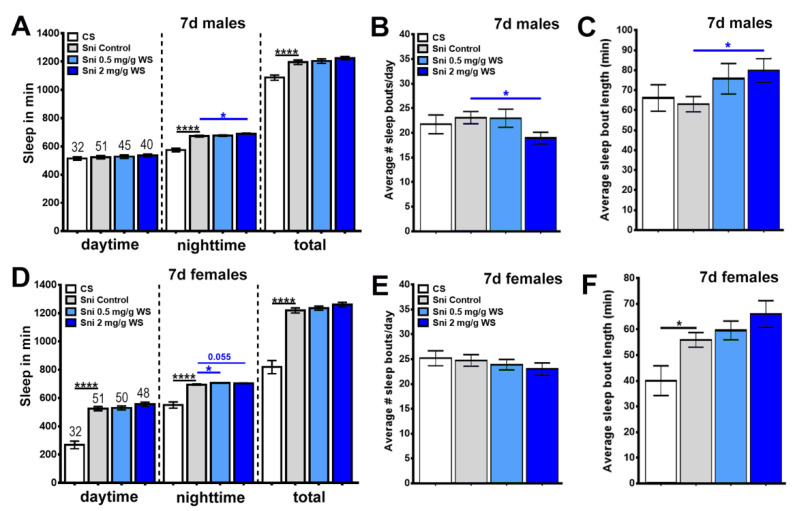
Average number of time asleep, sleep bouts per day and sleep bout length for *sniffer* flies treated with WS (0.5 mg/g and 2 mg/g). *Drosophila melanogaster sniffer* males (**A**–**C**) and females (**D**–**F**) were fed standard food or standard food supplemented with WS (0.5 mg/g or 2 mg/g) for 7 days. Flies aged 8 days were placed in the activity monitor for 9 days. Locomotor activity was analyzed once every minute for 7 days of light/dark (12 h:12 h LD). (**A**,**D**) Average time spent asleep during 12h daytime, 12h nighttime, and an entire 24h day. (**B**,**E**) Number of sleep bouts during a day. A sleep bout was regarded as a period of 5-min or more with no activity. (**C**,**F**) Average length of sleep bouts. The number of flies tested is indicated above the bars. Error bars represent SEM. * = *p* < 0.05, **** = *p* < 0.0001 compared to untreated *sniffer* flies (Sni control).

For this, we measured the average time spent asleep per day over a 7d period. When using male *sniffer* flies, we found no significant difference in the sleep time during daytime, either compared to CS or to WS treated flies (Figure 7A). During the night-time, however, significant differences were noted. CS flies had significantly less sleep than untreated male *sniffer* flies (Figure 7A). On the other hand, male *sniffer* flies treated with 2 mg/g WS had significantly more sleep during night-time compared to untreated *sniffer* flies (Figure 7A). When analyzing total sleep time, we still detected the differences in sleep time between CS and untreated male *sniffer* but the effects of WS treatment were not significant when combining daytime and night-time sleep (Figure 7A).

When analyzing the average number of sleep bouts per day, no difference was detected in male flies between CS, untreated *sniffer* flies, and *sniffer* flies treated with 0.5 mg/g WS. (Figure 7B). However, male *sniffer* flies treated with 2 mg/g WS had a significantly smaller average number of sleep bouts compared to untreated male *sniffer* flies indicating that sleep in this group was less fragmented (Figure 7B).

In examining the average sleep bout length of male flies, CS flies and untreated sniffer flies were indistinguishable and *sniffer* flies treated with 0.5 mg/g WS had a greater average than untreated *sniffer* flies (Figure 7C). However, this difference was not significant. In contrast, male *sniffer* flies treated with 2 mg/g WS had a significantly greater average sleep bout length than untreated *sniffer* flies (Figure 7C).

When analyzing female flies, we found that CS flies had significantly less sleep than untreated *sniffer* flies at all times (Figure 7D). There were no significant differences in sleep time during daytime or in total sleep time between untreated and *sniffer* flies treated with 0.5 mg/g and 2 mg/g WS (Figure 7D). As with males, WS treated female *sniffer* flies showed more sleep than untreated female sniffer flies (Figure 7D). Whereas this did reach significance when treated with 0.5 mg/g, there was no significant difference when treated with 2 mg/g WS although the difference tended towards significance with a *p*-value of 0.055 (Figure 7D).

In examining the average number of sleep bouts per day, we did not detect any significant differences. Although *sniffer* flies treated with 0.5 mg/g and 2 mg/g WS had lower averages than untreated female *sniffer* flies, none of these differences were significant (Figure 7E).

When measuring average sleep bout length in female flies, untreated *sniffer* flies had a greater average than CS flies and *sniffer* flies treated with 0.5 mg/g and 2 mg/g WS had greater averages than untreated female *sniffer* flies with the average value increasing with increasing dose. However, only the difference between female CS flies and untreated female *sniffer* flies was significant (Figure 7F).

## 4. Discussion

In this study, we investigated the behavioral effects of the water extracts of two plant materials, *W. somnifera* roots and *C. asiatica* aerial parts in the *D. melanogaster* mutant *sniffer* model which has been shown to have high levels of oxidative stress. Oxidative stress plays a role in neurodegeneration in normal [49] and pathological [4,50] aging and botanicals may potentially ameliorate this. While *sniffer* flies are not a model of aging, their high levels of oxidative stress can mimic that seen in aging. *Sniffer* flies show significantly impaired phototaxis response compared to wild type CS flies (Figure 1) and the phenotype gets worse with age (Figure 4). *Sniffer* flies are also a convenient model as they exhibit the high oxidative stress phenotype early in life compared to an aging model.

### 4.1. WS and Phototaxis

When treated with WS (0.5 mg/g and 2 mg/g) in standard food, *sniffer* flies saw an improvement in locomotion in phototaxis experiments for both male and female flies compared to *sniffer* control flies (Figure 6). The effect was dose dependent with 2 mg/g providing more protection than 0.5 mg/g whereby this was only significant for females when comparing the two doses directly. However, in both sexes, treatment with 2 mg/g resulted in *sniffer* flies reaching the level of locomotion seen in CS flies (Figure 6). In contrast, flies treated with 0.5 mg/g, although also showing significant improvement compared to untreated flies, did not reach CS levels.

Previous studies have also shown that *W. somnifera* improves locomotion in motor deficient *D. melanogaster* flies. Manjunath and Muralidhara found that standard *Drosophila* food supplemented with an undefined extract of the root of *W. somnifera* (0.005%, 0.01% and 0.05%) improved climbing in male *D. melanogaster* flies in a rotenone-induced model of neurotoxicity [51]. De Rose et al. also found that a methanolic extract of *W. somnifera* root (1%) improved locomotion in male flies in a *D. melanogaster* leucine-rich repeat kinase 2 (LRRK2) model of Parkinson’s Disease when the flies were aged 3–6 days or 10–15 days but not in flies aged 20–25 days [52]. In contrast to our studies, supplementation with *W. somnifera* failed to improve locomotion deficits to wild type levels in this model [52]. Other studies had contrasting findings. Jansen et al. found that *W. somnifera* root powder (23 mg/100 g *Drosophila* food) had no significant effect on locomotion in a loss of function phosphatase and tensin-induced putative kinase 1 (PINK1) *D. melanogaster* model of Parkinson’s disease or in wild type flies [53]. The contrasting effects of *W. somnifera* seen in these different studies may in part be due to the different preparations and doses used.

### 4.2. WS and Sleep

Sleep had not been assessed in *Sniffer* flies previously and therefore we first determined changes in the sleep pattern of these flies. To our surprise, our findings indicate that, overall, *sniffer* flies have more sleep than wild-type CS flies, and in males this increase is restricted to night-time sleep (Figure 7A–D). In both, males and females, treatment with WS further increased night-time sleep, suggesting that WS has a sleep promoting effect in *sniffer* flies even if they do not have a sleep deficit. Because we did not include treated CS flies, we cannot speculate if this is a general effect of WS on sleep in flies or specific for *sniffer* mutants. When analyzing the sleep pattern, we did not detect significant differences between untreated *sniffer* flies and CS controls (with the exception of increased sleep bout length in females) but in males, treatment with 2 mg/g WS decreased sleep bout number and increased sleep bout length. This indicates a less fragmented sleep pattern in treated flies, with them staying asleep for longer periods at a time and waking up less often. Again, we do not know whether this is a general effect of WS or only occurs when using *sniffer* flies.

Prior studies in a *Drosophila* model of Alzheimer’s disease overexpressing the human Aβ peptide reported that female flies treated with a hydroethanolic extract of WS (1 mg/mL) had increased total sleep time and decreased sleep latency but had no significant changes to sleep frequency and average time spent asleep [54]. The findings from our study and previous investigations indicate that sleep modulating compounds in *W. somnifera* can be extracted in an aqueous and aqueous/alcoholic solution and therefore both should be beneficial in supporting sleep. However, further investigations are needed to evaluate the sleep modulating properties of these extracts.

In contrast to our model, the Alzheimer model flies did have sleep deficits compared to controls and therefore the treatment directly improves this disease-associated phenotype. Due to the *sniffer* flies not having sleep deficits, and in fact sleeping more than CS flies, we do not think that the changes in sleep contribute to the phenotypes described in this mutant. Nevertheless, improving the sleep pattern may have a beneficial effect even if not directly addressing a deleterious phenotype.

### 4.3. CAW and Phototaxis

When CAW was mixed into standard *Drosophila* food or in yeast paste, we observed an improvement in phototaxis in *sniffer* flies although the effect was better when given in yeast. *D. melanogaster* occupies food sources which contain yeast and it has been shown experimentally that while *D. melanogaster* has middling attraction to unfermented, yeast-free substrates, flies have a strong preference for fermented, yeast-containing substrates [55]. Because of the stronger attraction to yeast-containing substrates, the *sniffer* flies may have consumed more of this substrate, and in turn more CAW, compared to standard *Drosophila* food.

In general, female *sniffer* flies showed more significant improvements in locomotion compared to males. Females showed an improvement in their performance when testing CAW in standard food and also when tested at an advanced stage (10 days of age), while males did not.

Investigations on the effect of derivatives of CAW on locomotion revealed an improvement in locomotion in both male and female *sniffer* flies treated with A1 (a subfraction of CAW) or a mix of CQAs found in A1 but not with TTs found in A1 (Figure 4). This suggests that the CQAs in CAW are responsible for the improved locomotion in *sniffer* flies observed with A1 and potentially also CAW. The improvement in locomotion from the A1 subfraction and caffeoylquinic acids may be due to an antioxidant effect. Previous in vitro studies with cell lines by Gray et al. found that treatment of the cells with both a water extract of *C. asiatica* as well as caffeoylquinic acids found in the water extract had an antioxidant effect [56]. Other in vitro studies by Liang et al. have also found that caffeoylquinic acids can scavenge free radicals [57].

When analyzing the phototaxis experiments, we found that female *sniffer* flies exhibited greater transition towards the light than male *sniffer* flies (Figure 2, Figure 3, Figure 4 and Figure 5). This may be due to sex differences in resistance to oxidative stress in *D. melanogaster*. A study by Niveditha et al. found that female *D. melanogaster* flies are more resistant to oxidative stress as they have lower levels of reactive oxygen species (ROS) and higher levels of antioxidant enzymes as they age compared to male flies [58]. Thus, female flies have a higher level of baseline resistance to oxidative stress and this may also be the case in the *sniffer* mutant.

*C. asiatica* has also been tested in other *Drosophila* models. In *D. melanogaster* models of Parkinson’s disease, the effects of *C. asiatica* have been mixed with Jansen et al. finding no improvement [53] and Siddique et al. finding significant improvement on locomotion with increased doses [59]. It must be noted that both studies used different *Drosophila* models of Parkinson’s disease as well as different preparations of *C. asiatica*. Jansen et al. used dried aerial parts of *C. asiatica* (7 mg/100 g food) in the PINK1 *Drosophila* fly model [53]. Siddique et al. used an acetone extract of *C. asiatica* leaves added to the diet at 0.25, 0.50 and 1.0 µL/mL in transgenic flies expressing normal human alpha synuclein (h-αS) [59]. Our results are more in agreement with the results from Siddique et al. that *C. asiatica* is protective but at fairly high doses.

## 5. Conclusions

Water extracts of *C. asaitica* and *W. somnifera* improved locomotion in the *sniffer Drosophila* model of oxidative stress. The *W. somnifera* water extract also improved night-time sleep for male and female *sniffer* flies and promoted less fragmented sleep in male *sniffer* flies. Exploration into other *Drosophila* models of ageing and age-related diseases as well as investigations with alcoholic extracts of *W. somnifera* and *C. asiatica* and their constituents are needed to provide more insight into the beneficial effects of these herbs.

## Figures and Tables

**Table 1:** Concentration of caffeoylquinic acids (CQAs) and triterpenes (TT) in *Centella asiatica* water extract (CAW), CAW subfraction A1, CQA mix and CAST^TM^. Fractionation of CAW using the scheme described in Section 2.2.3 resulted in a fraction (A1) where the CQAs and triterpene glycosides had been concentrated (on a % *w*/*w*) basis compared to their content in CAW. This is due to the removal of a large proportion of methanol insoluble (very polar) and dichloromethane (non-polar) components of CAW in earlier fractionation steps. nd = not detected.

Compound	Concentration of Compounds in Test Solutions (mg/mL) (10× That Present in *Drosophila* Food)			
	CAW 100 mg/mL	A1 11.35 mg/mL	CQA Mix	CAST 2 mg/mL
**CQAs:**				
**Neochlorogenic acid**	0.21	0.02	0.02	-
**Chlorogenic acid**	0.21	0.06	0.06	-
**Cryptochlorogenic acid**	0.1	0.02	0.02	-
**1,3-dicaffeoylquinic acid**	0.23	0.08	0.08	-
**1,5-dicaffeoylquinic acid**	0.53	0.50	0.50	
**Isochlorogenic acid A**	0.54	0.24	0.24	-
**Isochlorogenic acid B**	0.34	0.08	0.08	-
**Isochlorogenic acid C**	0.18	0.00	0.00	-
				
**Triterpenes:**				
**Asiatic acid**	nd	0.01	-	0.46
**Madecassic acid**	nd	0.03	-	0.69
**Asiaticoside**	2.1	0.79	-	0.74
**Madecassoside**	0.56	0.21	-	-

## Data Availability

The data presented in this study are available in this manuscript.
